# Recurrent aggressive angiomyxoma presented with perianal mass and typical imaging swirl sign

**DOI:** 10.1016/j.ijscr.2020.06.050

**Published:** 2020-06-12

**Authors:** Ahmed Abduljabbar, Mohammad Wazzan

**Affiliations:** King Abdul Aziz University, Radiology Department, Faculty of Medicine, P.O. Box 80215. Jeddah, 21589, Jeddah, Saudi Arabia

**Keywords:** Aggressive angiomyxoma, AAM, Pelvic tumor, Swirl sign

## Abstract

**Introduction:**

Aggressive angiomyxoma is a rare pelvic neoplasm that arises commonly in middle aged women and usually has delayed presentation due to its typically silent and slow growing behavior.

**Clinical presentation:**

A 47-year-old woman presented at our hospital with recurrent attacks of pelvic pain and a perianal bulge. Her past medical history was relevant due to previous en bloc resection of a right ovarian angiomyxoma. Current cross sectional imaging, including computed tomography and magnetic resonance imaging, was performed and showed evidence of local recurrence. Various available treatment options were offered to the patient.

**Discussion:**

The imaging features of the aggressive angiomyxoma were peculiar and showed a swirl and lamellate appearance in T1 and T2 weighted images.

**Conclusion:**

A locally aggressive tumor with benign histopathology and swirl sign in imaging is highly suggestive of aggressive angiomyxoma.

## Highlights

• Silent growing pelvic mass in middle age women with tendency to insinuate within the pelvis is commonly seen in aggressive angiomyxoma.
• The presence of swirl sign with the mass and striated appearance in T1 weighted and T2 weighted images are commonly seen with this tumour.
• Surgical intervention, radiotherapy, hormonal therapy and chemotherapy are valid treatment options.

## 1 Introduction

Aggressive angiomyxoma (AAM) is a rare type of locally aggressive pelvic tumor frequently occurring in women [1,2]; metastasis is rare and occurs early in the course of disease progression, if at all. This type of tumor was first reported in 1983 by Steeper and Rosai. Despite its well-known aggressive nature due to a high tendency to infiltrate and recur, this type of tumor is histologically benign. Microscopic analysis of such masses will show typical non aggressive cells in addition to spindle cells mixed with medium sized vessels [3]. The tumor is more common in women and is found to arise in pelvic organs including the bladder and uterus [2,4]. In this report, we discuss a case of recurrent pelviabdominal AAM which demonstrated a characteristic “swirled” sign. The work has been reported in line with the Surgical CAse REport criteria [5].

## 2 Case report

A 47-year-old woman visited our medical facility due to a perianal bulge and recurrent attacks of pelvic pain. Thirty-six months earlier she had presented with chronic pelvic pain and had undergone en bloc resection of a right ovarian angiomyxoma. The patient underwent an exploratory midline laparotomy under general anesthesia. Upon entering the abdominal cavity, a large soft pelvic mass was noticed filling the pelvis, which was adherent to the right adnexa right ureter, and right pelvic side-wall. Fortunately, the mass was not infiltrating the adjacent organs; therefore, it was successfully resected along with the right adnexa after a careful pelvic side-wall dissection. The uterus, left adnexa, and upper abdomen were all normal.

A review of the operative pathology slides prepared 36 months earlier revealed hypocellularity, spindle-shaped myxoid cells without atypia, and intermediate thickened wall vessels with few mast cells. Immunohistochemical marker analysis tests were positive for estrogen receptor, progesterone receptor, and desmin and locally positive for SMA as well as for CD34. The tumor had a myxoid stroma and variable-sized vascular channels with different kind of cells, include ovoid, stellate, and spindle-shaped cells with scant cytoplasm [2].

**Fig. 1:**
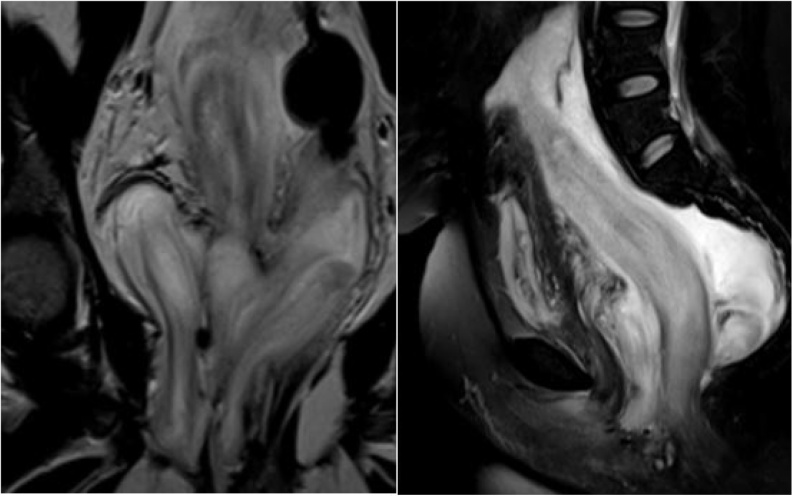
Coronal and sagittal T2 weighted fat saturated images of the pelvic region show a large pelvi-abdominal elongated mass measuring 24 × 18 × 16.5 cm in the maximum dimensions. It extends from the right ischioanal, ischiorectal fat planes and traverses the right levator ani muscle. Inferiorly, it is displacing the rectum anteriorly and to the left in addition to displacing the uterus and urinary bladder anteriorly. The mass is isointense to muscle on T1 WI and has a relatively high signal intensity in T2 WI with linear low signal bands (swirl appearance) noted within. No definitive restriction was noted in the diffusion image sequence. Post contrast images demonstrate heterogeneous enhancement throughout. The internal low-intensity “swirled” signal was observable on all image sequences.

**Fig. 2:**
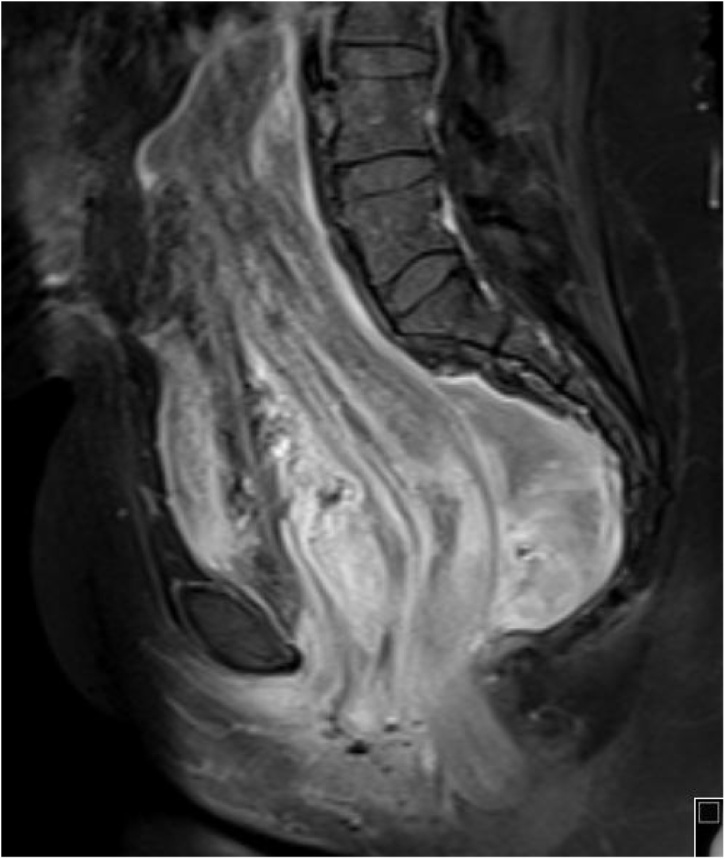
Sagittal T1 fat suppressed post contrast sequence shows the pelviabdominal mass demonstrating heterogeneous enhancement and the characteristic swirled appearance.

Current initial clinical assessment showed a partially defined, partially firm mass in the right perineal area. Cross sectional imaging revealed a large pelvi-abdominal, elongated, relatively homogenous hypo attenuated mass extending from the perineum to the mid abdomen. It measured 24 × 18 × 16.5 cm in the maximum dimensions, extending from the right ischioanal, ischiorectal fat planes and traversing the right levator ani muscle. Inferiorly, it had a mass effect on the rectum anteriorly and to the left as well as on other pelvic organs, mainly the urinary bladder (Fig. 1). The mass demonstrated iso intense signal intensity as compared to muscle in T1 weighted imaging (WI) sequences and relative brightness in T2WI; furthermore, alternating linear low signal bands (swirl appearance) were noted within the mass. There was no definitive restriction in the diffusion weighted image sequence. Post contrast images demonstrated heterogeneous enhancement throughout with linear hypointensity “swirled” signals (Fig. 2). The current imaging findings were pathognomonic for AAM and continued imaging follow up was final plane in accordance with patient preference.

## 3 Discussion

AAM is a rare pelvic tumor that grows slowly and mainly breaches the pelvic structures adjacent to its site of origin. Clinically, it is usually larger than 10 cm in size, arises from mesenchymal tissue, exhibits pathognomonic imaging features, and has a growth pattern that commonly involves the perineum and pelvic organs [6]. In women, it mostly involves the genital organs, including the labial, ovarian, or vulval mass or perineal hernias. The pattern of this disease indicates an increased propensity to occur in women, with a women to men prevalence ratio of approximately 6:1. This kind of tumor typically does not involve pediatric or geriatric patients and predominantly arises in the reproductive age group [7]. Displacement of the adjacent organs is more common than invasion, in addition to a high chance of recurrence after surgical removal, reported as approximately 83% by Steeper and Rosai and 47% by Chan et al. [3]. The recurrence rate is very high in the first few years, reaching up to 71% [8].

The high recurrence rate is attributed to soft tissue mass extension, and it is often not accurately documented due to its position in the pelvis between multiple organs, where tumor extension is seen above and below the urogenital and gastrointestinal organs; this also leads to incomplete resection and a high probability of residual tumor cells [3,9]. The clinical presentation varies from silent presentation versus slow growing, soft, and mobile pararectal or paravaginal masses which might be mistaken for pedunculated fibroid or hernia [10]. The imaging features of AAM on computed tomography typically indicate a circumscribed hypo-attenuated soft tissue mass as compared to musculature density [11].

Accurate preoperative assessment and detailed imaging analysis is crucial for treatment planning and also affects the selection of appropriate surgical treatment approaches—either perineal, abdominal, or both—to avoid residual tumor tissue in the surgical bed. Postoperative imaging is also mandatory to assess the usefulness of the surgery and to exclude recurrent disease or residual tumor.

On magnetic resonance imaging (MRI), it usually presents with hyperintense T2 signal intensity secondary to rich fluid content and low intensity in T1WI; the signal intensity of the mass is similar to that of muscle [[11], [12], [13]]. Pathognomonically, it demonstrates a laminated “swirled” linear hypointense signal intensity on T1WI and T2WI, which was also observed in our case [[11], [12], [13]]. In dynamic post contrast imaging, there is heterogeneous avid enhancement secondary to the presence of high vascularity [14].

On gross assessment, the tumors usually present without a distinct outline and infiltrate the adjacent soft tissue, which has a grey to pink coloration and a rubbery, gelatinous surface. Histologically, they have low cellularity, cells with regular nuclei and absence of atypia or mitotic changes, and an edematous stroma with numerous blood vessels.

Different immunohistochemically-detectable receptors can be observed in abdominopelvic pathologies, and analyzing the expression of these receptors is important for selecting the appropriate medical treatment. In this case report, the focus was on estrogen and progesterone receptors [15]; other immunostaining markers that aid in reaching the most likely diagnosis and in narrowing the differential diagnoses include desmin, actins, and CD34 [15]. Chromosomal translocations involving 12q13–15 have been reported in several neoplasms, including aggressive angiomyxomas [16]. Pelvic masses are commonly encountered in female patients, and multiple alternative diagnoses can be considered when AAM is suspected, such as neurogenic, fat containing with myxoid type, and multiple other types of myxoid tumors [17].

The treatment options for AAM are varied, and a multidisciplinary approach, including wide local excision, preoperative embolization, hormonal therapy, and to a lesser extent chemo/radiotherapy, may be used [18,19]. Surgical treatment is widely accepted to be a radical option in the treatment. Hormonal therapy with gonadotropin-releasing hormone analogs, raloxifene, and tamoxifen are used to decrease the tumor size in order to help surgeons perform complete surgical resection and treat recurrent disease [13].

## 4 Conclusion

AAMs are rare, histologically benign tumors with high potential for local infiltration to adjacent organs and show a unique characteristic “swirl sign” seen on MRI. A variety of treatment options are available to patients according to tumor stage, and subsequent long-term imaging is advised to exclude the possibility of commonly observed local recurrence.

## Declaration of Competing Interest

None.

## Sources of funding

None.

## Ethical approval

Available.

## Consent

Written informed consent was obtained from the patient for publication of this case report and accompanying images. A copy of the written consent is available for review by the Editor-in-Chief of this journal on request.

## Author contribution

Ahmed Abduljabbar (Writing the manuscript abstract and introduction, editing the manuscript and submission process).

Mohammad Wazzan (Writing the manuscript case report, discussion, select the figures and related details, revising the final manuscript).

## Registration of research studies

None.

## Guarantor

Ahmed Abduljabbar.

## Provenance and peer review

Not commissioned, externally peer-reviewed.
